# Preoperative Risk Score Predicting 90-Day Mortality After Liver Resection in a Population-Based Study

**DOI:** 10.1097/MD.0000000000000059

**Published:** 2014-09-05

**Authors:** Chun-Ming Chang, Wen-Yao Yin, Yu-Chieh Su, Chang-Kao Wei, Cheng-Hung Lee, Shiun-Yang Juang, Yi-Ting Chen, Jin-Cherng Chen, Ching-Chih Lee

**Affiliations:** Department of Surgery (C-MC, W-YY, C-KW, C-HL, J-CC); Department of Otolaryngology (C-CL); Center for Clinical Epidemiology and Biostatistics (S-YJ, C-CL); Division of Hematology-Oncology, Department of Internal Medicine (Y-CS); Cancer Center (Y-CS, C-CL), Dalin Tzu Chi Hospital, Buddhist Tzu Chi Medical Foundation, Chiayi; School of Medicine, Tzu Chi University, Hualian (C-MC, W-YY, C-KW, C-HL, J-CC, C-CL); and Department of Computer Science and Information Engineering, National Cheng Kung University, Tainan City (Y-TC), Taiwan.

## Abstract

The impact of important preexisting comorbidities, such as liver and renal disease, on the outcome of liver resection remains unclear. Identification of patients at risk of mortality will aid in improving preoperative preparations. The purpose of this study is to develop and validate a population-based score based on available preoperative and predictable parameters predicting 90-day mortality after liver resection using data from a hepatitis endemic country.

We identified 13,159 patients who underwent liver resection between 2002 and 2006 in the Taiwan National Health Insurance Research Database. In a randomly selected half of the total patients, multivariate logistic regression analysis was used to develop a prediction score for estimating the risk of 90-day mortality by patient demographics, preoperative liver disease and comorbidities, indication for surgery, and procedure type. The score was validated with the remaining half of the patients.

Overall 90-day mortality was 3.9%. Predictive characteristics included in the model were age, preexisting cirrhosis-related complications, ischemic heart disease, heart failure, cerebrovascular disease, renal disease, malignancy, and procedure type. Four risk groups were stratified by mortality scores of 1.1%, 2.2%, 7.7%, and 15%. Preexisting renal disease and cirrhosis-related complications were the strongest predictors. The score discriminated well in both the derivation and validation sets with c-statistics of 0.75 and 0.75, respectively.

This population-based score could identify patients at risk of 90-day mortality before liver resection. Preexisting renal disease and cirrhosis-related complications had the strongest influence on mortality. This score enables preoperative risk stratification, decision-making, quality assessment, and counseling for individual patients.

## INTRODUCTION

Liver surgery is a more common procedure that is performed at numerous hospitals worldwide.^1^ In Asia, the prevalence of hepatitis and hepatocellular carcinoma (HCC) is high.^2^ Because donor shortage remains a major problem in Asia, liver resection is considered the first-line treatment for some patients with HCC.^3,4^ In addition, intrahepatic cholangiocarcinoma, metastatic malignancies, and benign diseases such as trauma, intrahepatic bile duct stones, and benign tumors also require liver resection.^1^

With the advance in management of liver diseases and surgical techniques, the mortality rate of liver resection has decreased to <5% during the last 2 decades in most specialized centers^5,6^; however, patient safety remains of utmost concern. As the indications for liver resection expand and the number of liver resections increases worldwide, preoperative stratification of at-risk patients is needed to improve preoperative evaluation and preparation. Some investigators have developed scores to predict mortality after liver resection.^7–11^ However, a limitation of the available studies and those addressing prediction scores is that they included postoperative parameters and did not preoperatively stratify patients at risk of mortality. Furthermore, these studies were conducted in specialized centers.^9,10^ For scores derived from administrative data,^7,8,11^ the majority of their records indicated a diagnosis of metastatic disease, and noncirrhosis patients indicated for hepatic resection. It is not clear whether these scores are applicable to patients with hepatitis and cirrhosis in endemic areas.

Therefore, by accounting for prognostic preoperative factors, the purpose of this study was to develop and validate a simple, applicable score based on available preoperative and predictable parameters such as hepatitis/cirrhosis, preexisting cirrhosis-related complications, major comorbidities, and the magnitude of liver resection to predict 90-day mortality in patients scheduled for liver resection. Specifically, we sought to identify preoperative factors associated with 90-day mortality. To make the score as close as possible to the daily practice, we considered parameters readily available in the preoperative assessment. Unpredictable intraoperative factors such as duration of surgery, ischemic time, and blood loss were not considered. We used a population-based national database of Taiwanese patients who underwent liver resection between 2002 and 2006 to develop and validate this predictive model.

## PATIENTS AND METHODS

### Ethics Statements

This study was initiated after receiving approval from the Institutional Review Board of the Buddhist Dalin Tzu Chi General Hospital, Taiwan. Because the identification numbers and personal information of the individuals included in the study were not included in the secondary files, the review board stated that written patient consent was not required.

### Patients and Study Design

We used data from 2002 to 2006 from the National Health Insurance Research Database (NHIRD) that covers medical benefit claims for >23 million people in Taiwan (approximately 99% of Taiwan’s population).^12^ Taiwan’s National Health Insurance (NHI) provides universal insurance coverage, comprehensive services, and is a single-payer system with the government as the sole insurer. The database was monitored for completeness and accuracy by Taiwan’s Department of Health. Patients who underwent liver resection for cancer disease (HCC, cholangiocarcinoma, and metastatic malignancy), and benign disease (eg, trauma, intrahepatic bile duct stones, and benign tumors) between 2002 and 2006 were included. A total of 13,159 patients were identified. Patient mortalities were identified from the National Register of Deaths Database.

### Measurements

The primary outcome measure was 90-day mortality, defined as death from all causes occurring after liver resection. The predictor variables were patient age, sex, indication for surgery, comorbidity, and surgical procedure (major, lobectomy or more; minor, less than lobectomy). The comorbidities included hypertension, ischemic heart disease, arrhythmia, cerebrovascular disease, chronic obstructive pulmonary disease, and associated conditions including chronic bronchitis and asthma, diabetes, hyperlipidemia, and renal diseases including acute and chronic renal failure, glomerulonephritis, and nephrotic syndrome. Patients’ severity of underlying liver disease was evaluated by the presence or absence of hepatitis/cirrhosis and cirrhosis-related complications. The cirrhosis-related complications included portal hypertension, esophageal/gastric variceal bleeding, ascites, pleural effusion, encephalopathy, and hepatorenal syndrome. We identified the presence or absence of these preexisting comorbidities and cirrhosis-related complications in each patient by querying the Taiwan NHI database using the *International Classification of Diseases, Ninth Revision* (*ICD-9*) codes. Any preoperatively existing comorbidity or cirrhosis-related complication was identified from the *ICD-9* codes for inpatients within 6 months before surgery. For outpatients, the diseases that were coded 3 times or more in the records within 6 months before surgery were also identified from the *ICD-9* codes.

### Statistical Analysis

The SPSS (version 15; SPSS Inc, Chicago, IL) were used for data analysis. Univariate analyses were performed for the predictive variables. Those with statistical significance at the level of *P* < 0.1 were entered into a logistic regression model for odd ratios of 90-day mortality; multivariate logistic regression analysis was used to calculate the risk of mortality after adjustments for variables. Then, we developed a model to predict 90-day mortality in a randomly selected half of our study population (derivation population) and validated the model in the remaining half (validation population). For the random selection of patients for the derivation and validation populations, the incidences of mortality were comparable in both populations (defined as the incidence for the whole population). The β coefficients from the logistic regression model were used to develop an integer-based weighted point system for stratifying 90-day mortality risk. The referent for each variable was assigned a value of 0 and the coefficients for the others were adjusted proportionally, rounding to the nearest integer. Individual scores were assigned by summing the individual risk factor points. The risk scores were stratified into 4 risk groups as follows: score of 0 to 1, score of 2 to 3, score of 4 to 5, and score of 6 and above.

Within the derivation set, the risk score was calculated for each patient, and discrimination was assessed using the area under the receiver operating characteristic (ROC) curve. For validation of the risk score, the remaining half of the random sample was used. The risk score model was applied, and discrimination was assessed by ROC curve analysis.

## RESULTS

A total of 13,159 patients who underwent liver resection between 2002 and 2006 were identified from the NHIRD. The patient characteristics are summarized in Table 1. The 90-day mortality rate for the overall cohort was 3.9%. Up to 39.2% of the patients’ records indicated a diagnosis of hepatitis and cirrhosis. The majority (61.2%) of liver resections were performed for HCC. Minor resection (less than lobectomy) was performed primarily (75.8%). After randomly sampling this group, the derivation set contained 6580 patients and the validation set contained 6579 patients.

**TABLE 1:**
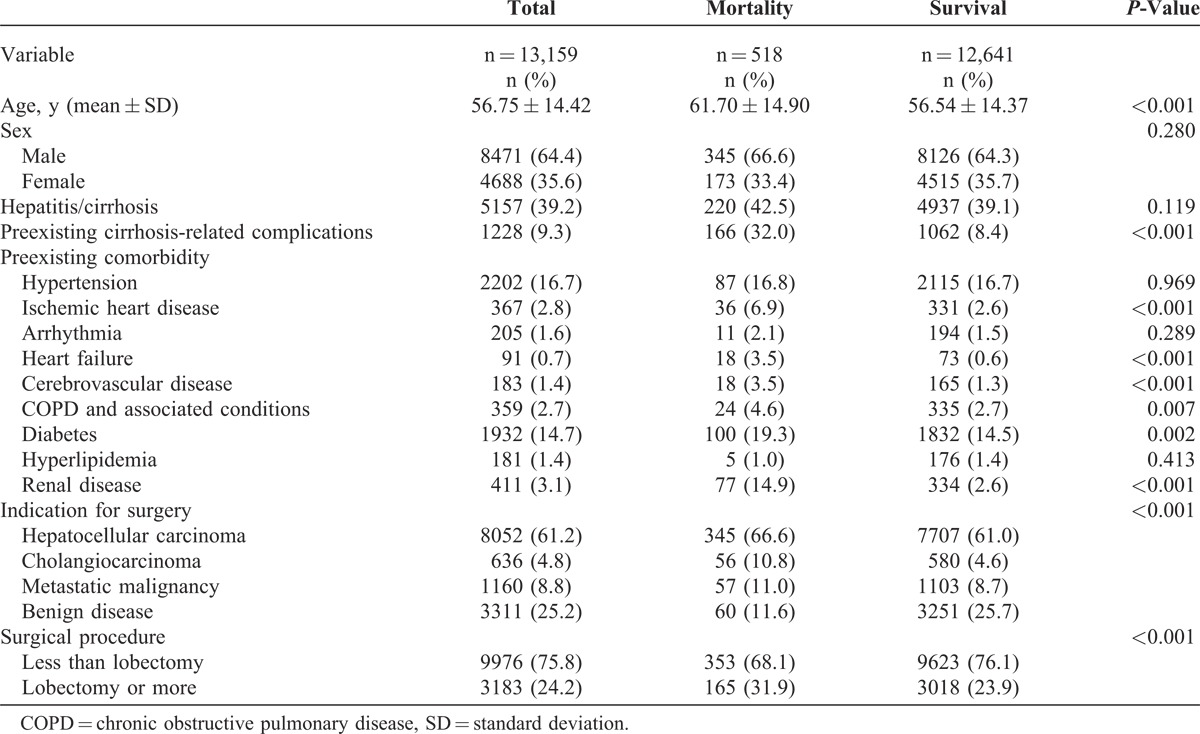
Baseline Patient Characteristics

Table 2 shows the results of the multivariate logistic regression analysis. After adjusting for other factors, among those preexisting comorbidities, renal disease showed the strongest association with 90-day mortality (adjusted odds ratio, 5.29; 95% confidence interval, 3.98–7.03; *P* < 0.001), followed by cirrhosis-related complications (adjusted odds ratio, 4.70; 95% confidence interval, 3.83–5.76; *P* < 0.001), heart failure (adjusted odds ratio, 2.92; 95% confidence interval, 1.61–5.29; *P* < 0.001), cerebrovascular disease (adjusted odds ratio, 2.17; 95% confidence interval, 1.29–3.66 ; *P* = 0.004), and ischemic heart disease (adjusted odds ratio, 1.98; 95% confidence interval, 1.34–2.92; *P* = 0.001). Cholangiocarcinoma (adjusted odds ratio, 1.73; 95% confidence interval, 1.26–2.37; *P* = 0.001) and metastatic malignancy (adjusted odds ratio, 1.44; 95% confidence interval, 1.07–1.93; *P* = 0.017) showed stronger association with 90-day mortality than HCC. Old age (adjusted odds ratio, 1.70; 95% confidence interval, 1.41–2.05; *P* < 0.001) and major procedure (lobectomy or more) (adjusted odds ratio, 1.44; 95% confidence interval, 1.17–1.76; *P* < 0.001) were also associated with 90-day mortality.

**TABLE 2:**
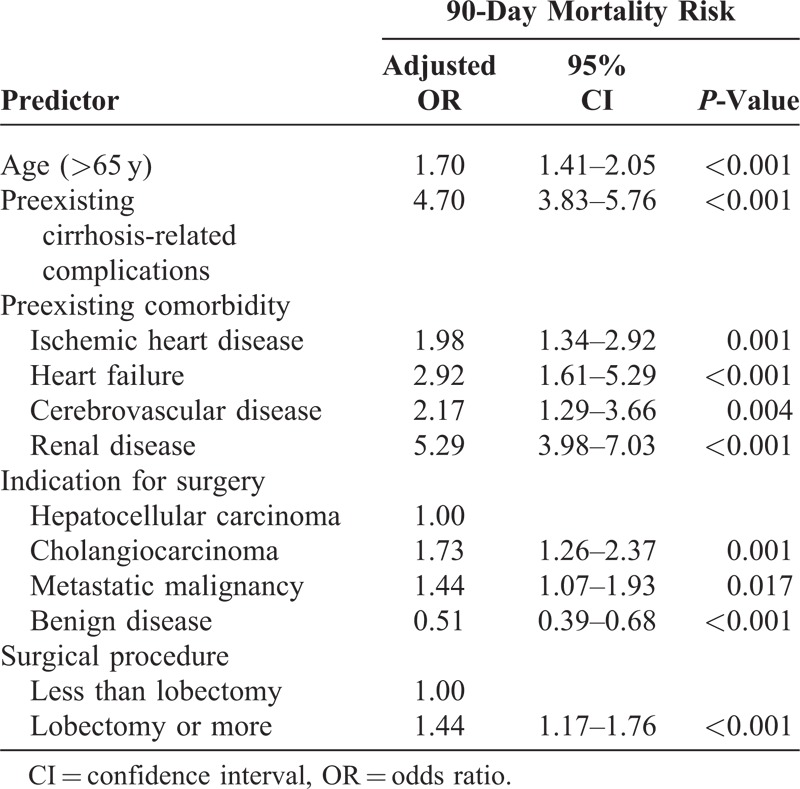
Multivariate Logistic Regression Analysis

**FIGURE 1:**
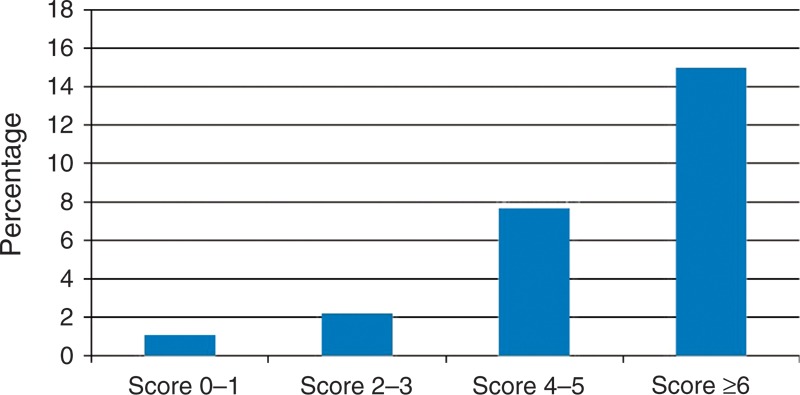
Estimated risk of 90-day mortality by prediction score group.

We further grouped the indications for surgery into benign and malignant diseases to reduce the number of variables for the prediction model. Table 3 shows the results of the multivariate model with the predictors and risk scores from the 6580 patients randomly selected for the development of the model. Grouping the scores into 4 risk groups resulted in a gradient for mortality estimates. The mortality rates of each group were 1.1%, 2.2%, 7.7%, and 15% (Figure 1). The model discriminated the derivation set well, with an area under the ROC curve of 0.75. When applied to the validation set, the model continued to discriminate well, with an area under the ROC curve of 0.75.

**TABLE 3:**
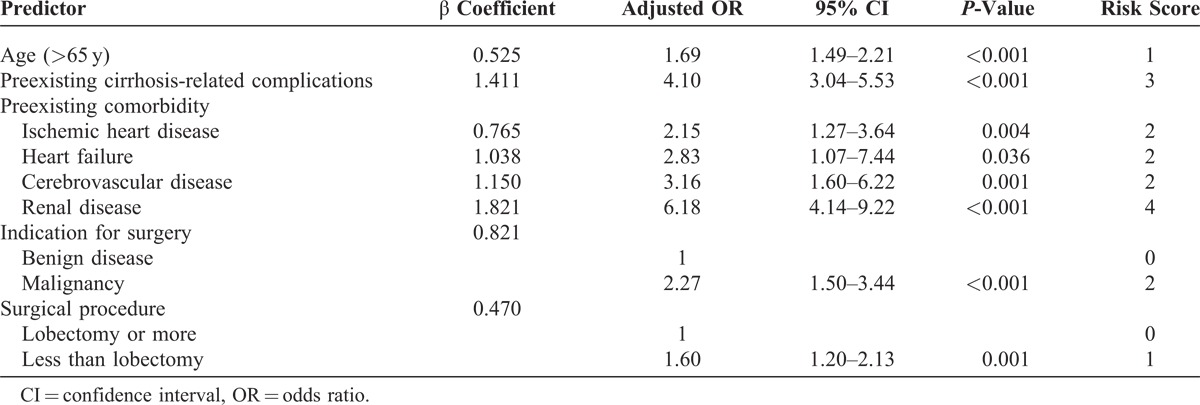
Development of a Predictor Score Based on Multivariate Logistic Regression Analysis of the Derivation Set

## DISCUSSION

In this study from a national database identifying 13,159 liver resections between 2002 and 2006 in Taiwan, we developed and validated an easily applicable score to predict the risk of perioperative mortality in patients scheduled for hepatic resection. The overall perioperative mortality was 3.9%, whereas the mortality according to score groups ranged from 1.1% to 15%. Factors most strongly associated with 90-day mortality were preexisting renal disease and cirrhosis-related complications.

The strength of this study is that it was a nationwide population-based study including almost all the patients undergoing hepatic resection in Taiwan. By the end of 2006, the NHI covered 99.0% of Taiwan’s population,^12^ with nearly complete follow-up information of mortality among the entire study population, and the dataset was routinely monitored for diagnostic accuracy by the NHI Bureau of Taiwan. The present score is unlike the population-based score published by Simons et al^7,11^ using the Charlson Comorbidity Index score to classify patients. Their data were queried from patient discharges for hepatic resection. It was unclear whether these comorbidities, particularly liver and renal disease, the 2 main concerns influencing the outcome of hepatic resection, were present before or occurred after the surgery. In our study, the entire dataset was queried from preoperative inpatient and outpatient records, using only the clinical factors that were widely available before surgery. Moreover, the aforementioned previous study focused on predicting inhospital mortality as the outcome measure, which does not reflect all deaths that occur after discharge. In contrast, we measured 90-day mortality to account for all risk during the perioperative period.

Increasingly, more liver resections are being performed on patients who may be at higher risk given the nature of liver diseases such as cirrhosis, and previous investigators have consistently suggested that careful patient selection is crucial for achieving better outcomes.^13,14^ The main preoperative factors that predict postoperative liver dysfunction include the presence of chronic liver disease, extensive liver resection, and the presence of a small liver remnant. It has been reported that portal hypertension is not a contraindication for hepatic surgery in properly selected patients, who had similar survival compared with patients without portal hypertension.^15,16^ However, the present population-based findings derived from patients who underwent liver resection supported the notion that preexisting cirrhosis-related complications remain a significant influence on increasing the risk of perioperative mortality.

Acute renal failure is a major complication after abdominal surgery that is associated with major morbidity and mortality.^17,18^ However, very limited data exist regarding the impact of renal disease on the outcomes of liver resection.^19,20^ Some single-center studies showed that end-stage renal disease patients undergoing liver resection did not have higher perioperative mortality compared with patients without end-stage renal disease.^21–23^ Our findings revealed that among the factors associated with perioperative mortality because of liver resection, preexisting renal disease was the strongest, which was also in agreement with previous studies of other procedures showing that postoperative acute renal failure significantly impaired the outcomes after major surgery.^24,25^ This result indicates that when treating patients with renal disease, careful surgical techniques and perioperative management are crucial for lower mortality risk.

There are some limitations in this study. First, patient diagnosis and identification of comorbidities were completely dependent on *ICD-9* codes, and any coding errors in patients’ underlying diseases could lead to disparities in preexisting comorbidities and cirrhosis-related complications. Nonetheless, the NHI Bureau of Taiwan randomly reviews the charts and interviews patients in order to verify diagnostic accuracy.^26^ Second, the NHIRD is an administrative database and lacks certain clinical information such as the subtleties of liver dysfunction, the glomerular filtration rate, and the creatinine for renal disease that may not be completely captured using *ICD-9* codes alone. However, the diagnosis of hepatitis/cirrhosis, preexisting cirrhosis-related complications, and renal disease represented the comorbidity status of liver and renal disease before surgery. Further studies with primary data might be conducted to reveal a deeper insight about the severity of these preexisting diseases and their relationship with perioperative mortality. Third, instead of surgical mortality, all-cause mortality was used. However, the short-term surgical and all-cause mortality differed only to a small degree.

Despite these limitations, our score is an easy to use tool for clinical application with good discriminatory ability. The use of a national database that included almost all liver resections increased its generalizability over many single-institution case series. As the experience in liver resections increases, the number of patients with more and worse comorbidities being considered for liver resection may increase. In addition to the widely used Child–Turcotte–Pugh score in liver surgery, the incorporation of preexisting comorbidities and cirrhosis-related complications in the present tool might be helpful for patient counseling and informed consent. This type of risk stratification also allows for different treatment options to be considered in patients with certain comorbidities.

In summary, our results demonstrated that this score could be used to identify patients at higher risk of 90-day mortality before liver resection. This population-based risk score also revealed that preexisting renal and cirrhosis-related complications had the strongest influence on 90-day mortality in liver resection. This score may be useful for preoperative risk stratification, decision-making, quality assessment, and counseling of individual patients.

## ACKNOWLEDGMENTS

This study is based in part on data from the NHIRD provided by the Bureau of National Health Insurance, Department of Health and managed by the National Health Research Institutes (*registry* number 101115). The interpretation and conclusions contained herein do not represent the opinions of the Bureau of National Health Insurance, Department of Health, or the National Health Research Institutes.

## References

[1] Justin B Dimick M Reid M National trends in the use and outcomes of hepatic resection. J Am Coll Surg. 2004;199:31–38. 15217626 10.1016/j.jamcollsurg.2004.03.005

[2] Tanaka M Katayama F Kato H Hepatitis B and C virus infection and hepatocellular carcinoma in China: a review of epidemiology and control measures. J Epidemiol. 21:401–416. 22041528 10.2188/jea.JE20100190 PMC3899457

[3] Mazzaferro V Regalia E Doci R Liver transplantation for the treatment of small hepatocellular carcinomas in patients with cirrhosis. N Engl J Med. 1996;334:693–699. 8594428 10.1056/NEJM199603143341104

[4] Bigourdan JM Jaeck D Meyer N Small hepatocellular carcinoma in Child A cirrhotic patients: hepatic resection versus transplantation. Liver Transpl. 2003;9:513–520. 12740796 10.1053/jlts.2003.50070

[5] Belghiti J Hiramatsu K Benoist S Seven hundred forty-seven hepatectomies in the 1990s: an update to evaluate the actual risk of liver resection. J Am Coll Surg. 2000;191:38–46. 10898182 10.1016/s1072-7515(00)00261-1

[6] Dimick JB Cowan JA Jr Knol JA Hepatic resection in the United States: indications, outcomes, and hospital procedural volumes from a nationally representative database. Arch Surg. 2003;138:185–191. 12578418 10.1001/archsurg.138.2.185

[7] Simons JP Hill JS Ng SC Perioperative mortality for management of hepatic neoplasm: a simple risk score. Ann Surg. 2009;250:929–934. 19855257 10.1097/SLA.0b013e3181bc9c2f

[8] Simons JP Ng SC Hill JS In-hospital mortality for liver resection for metastases: a simple risk score. J Surg Res. 2009;156:21–25. 19577250 10.1016/j.jss.2009.03.073

[9] Balzan S Belghiti J Farges O The “50-50 criteria” on postoperative day 5: an accurate predictor of liver failure and death after hepatectomy. Ann Surg. 2005;242:824–828. 16327492 10.1097/01.sla.0000189131.90876.9e PMC1409891

[10] Hyder O Pulitano C Firoozmand A A risk model to predict 90-day mortality among patients undergoing hepatic resection. J Am Coll Surg. 2013;216:1049–1056. 23478548 10.1016/j.jamcollsurg.2013.01.004 PMC3985272

[11] Simons JP Ng SC Hill JS In-hospital mortality from liver resection for hepatocellular carcinoma: a simple risk score. Cancer. 2010;116:1733–1738. 20143433 10.1002/cncr.24904

[12] National Health Insurance Administration, Ministry of Health and Welfare, Taiwan. http://www.nhi.gov.tw/webdata/webdata.aspx?menu=17&menu_id=1023&WD_ID=1023&webdata_id=815 Accessed May 15, 2014.

[13] Song TJ Ip EW Fong Y Hepatocellular carcinoma: current surgical management. Gastroenterology. 2004;127 (5 suppl 1):S248–S260. 15508091 10.1053/j.gastro.2004.09.039

[14] Rahbari NN Mehrabi A Mollberg NM Hepatocellular carcinoma: current management and perspectives for the future. Ann Surg. 2011;253:453–469. 21263310 10.1097/SLA.0b013e31820d944f

[15] Cucchetti A Ercolani G Vivarelli M Is portal hypertension a contraindication to hepatic resection? Ann Surg. 2009;250:922–928. 19855258 10.1097/SLA.0b013e3181b977a5

[16] Santambrogio R Kluger MD Costa M Hepatic resection for hepatocellular carcinoma in patients with Child-Pugh’s A cirrhosis: is clinical evidence of portal hypertension a contraindication? HPB (Oxford). 2013;15:78–84. 23216782 10.1111/j.1477-2574.2012.00594.x PMC3533715

[17] Kheterpal S Tremper KK Englesbe MJ Predictors of postoperative acute renal failure after noncardiac surgery in patients with previously normal renal function. Anesthesiology. 2007;107:892–902. 18043057 10.1097/01.anes.0000290588.29668.38

[18] Bihorac A Yavas S Subbiah S Long-term risk of mortality and acute kidney injury during hospitalization after major surgery. Ann Surg. 2009;249:851–858. 19387314 10.1097/SLA.0b013e3181a40a0b

[19] Poon RT Fan ST Lo CM Improving perioperative outcome expands the role of hepatectomy in management of benign and malignant hepatobiliary diseases: analysis of 1222 consecutive patients from a prospective database. Ann Surg. 2004;240:698–708. 15383797 10.1097/01.sla.0000141195.66155.0c PMC1356471

[20] Saner F Kidney failure following liver resection. Transplant Proc. 2008;40:1221–1224. 18555153 10.1016/j.transproceed.2008.03.068

[21] Cheng SB Wu CC Shu KH Liver resection for hepatocellular carcinoma in patients with end-stage renal failure. J Surg Oncol. 2001;78:241–246. 11745817 10.1002/jso.1160

[22] Yeh CN Lee WC Chen MF Hepatic resection for hepatocellular carcinoma in end-stage renal disease patients: two decades of experience at Chang Gung Memorial Hospital. World J Gastroenterol. 2005;11:2067–2071. 15810070 10.3748/wjg.v11.i14.20 PMC4305773

[23] Orii T Takayama T Haga I Efficacy of a liver resection for hepatocellular carcinoma in patients with chronic renal failure. Surg Today. 2008;38:329–334. 18368322 10.1007/s00595-007-3634-1

[24] Kim CS Oak CY Kim HY Incidence, predictive factors, and clinical outcomes of acute kidney injury after gastric surgery for gastric cancer. PLoS One. 2013;8:e82289. 24349249 10.1371/journal.pone.0082289 PMC3857284

[25] Mooney JF Ranasinghe I Chow CK Preoperative estimates of glomerular filtration rate as predictors of outcome after surgery: a systematic review and meta-analysis. Anesthesiology. 2013;118:809–824. 23377223 10.1097/ALN.0b013e318287b72c

[26] National Health Insurance Administration, Ministry of Health and Welfare, Taiwan. http://www.nhi.gov.tw/webdata/webdata.aspx?menu=17&menu_id=659&webdata_id=4524 Accessed May 15, 2014.

